# Optimal respiratory-gated [^18^F]FDG PET/CT significantly impacts the quantification of metabolic parameters and their correlation with overall survival in patients with pancreatic ductal adenocarcinoma

**DOI:** 10.1186/s13550-019-0492-y

**Published:** 2019-03-13

**Authors:** Esther M. M. Smeets, Dominique S. Withaar, Willem Grootjans, John J. Hermans, Kees van Laarhoven, Lioe-Fee de Geus-Oei, Martin Gotthardt, Erik H. J. G. Aarntzen

**Affiliations:** 1 Department of Radiology and Nuclear Medicine, Radboud University Medical Center, Geert Grooteplein-Zuid 10, PO Box 9101, 6500 HB Nijmegen, The Netherlands; 2 Department of Radiology, Medisch Spectrum Twente, Enschede, The Netherlands; 3 Department of Surgery, Radboud University Medical Center, Nijmegen, The Netherlands; 4 Department of Radiology, Leiden University Medical Center, Leiden, The Netherlands; 5 Biomedical Photonic Imaging Group, University of Twente, Enschede, The Netherlands

**Keywords:** Optimal respiratory gating, [^18^F]FDG PET/CT, Pancreatic ductal adenocarcinoma, Metabolic parameters, Texture features

## Abstract

**Purpose:**

Metabolic parameters are increasingly being used to characterize tumors. Motion artifacts due to patient respiration introduce uncertainties in quantification of metabolic parameters during positron emission tomography (PET) image acquisition. The present study investigates the impact of amplitude-based optimal respiratory gating (ORG) on quantification of PET-derived image features in patients with pancreatic ductal adenocarcinoma (PDAC), in correlation with overall survival (OS).

**Methods:**

Sixty-nine patients with histologically proven primary PDAC underwent 2′-deoxy-2′-[^18^F]fluoroglucose ([^18^F]FDG) PET/CT imaging during diagnostic work-up. Standard image acquisition and reconstruction was performed in accordance with the EANM guidelines and ORG images were reconstructed with a duty cycle of 35%. PET-derived image features, including standard parameters, first- and second-order texture features, were calculated from the standard and corresponding ORG images, and correlation with OS was assessed.

**Results:**

ORG significantly impacts the quantification of nearly all features; values of single-voxel parameters (e.g., SUV_max_) showed a wider range, volume-based parameters (e.g., SUV_mean_) were reduced, and texture features were significantly changed. After correction for motion artifacts using ORG, some features that describe intra-tumoral heterogeneity were more strongly correlated to OS.

**Conclusions:**

Correction for respiratory motion artifacts using ORG impacts the quantification of metabolic parameters in PDAC lesions. The correlation of metabolic parameters with OS was significantly affected, in particular parameters that describe intra-tumor heterogeneity. Therefore, interpretation of single-voxel or average metabolic parameters in relation to clinical outcome should be done cautiously. Furthermore, ORG is a valuable tool to improve quantification of intra-tumoral heterogeneity in PDAC.

**Electronic supplementary material:**

The online version of this article (10.1186/s13550-019-0492-y) contains supplementary material, which is available to authorized users.

## Introduction

Pancreatic malignancies have a dismal prognosis. The primary treatment for patients with a resectable tumor is surgery, resulting in a 23% 5-year survival. In patients with unresectable tumor or metastatic disease, 5-year survival decreases to 6% [1]. Although FOLFIRINOX [2] and nab-paclitaxel [3] have shown slightly better outcome as compared to gemcitabine-containing regimen, no effective systemic treatment options are available for irresectable or distant metastatic disease [4].

2′-deoxy-2′-[^18^F]fluoroglucose ([^18^F]FDG) PET/CT scanning is not standard in the diagnostic work-up for pancreatic cancer. In most institutes, this modality is mainly used to rule out distant metastases prior to planned surgery. Recent literature suggests that metabolic features of the primary tumor might associate with overall survival (OS) in patients with pancreatic ductal adenocarcinoma (PDAC). For many other malignancies, e.g., endometrial cancer [5], non-small cell lung carcinoma [6, 7], sarcomas [8] and bone metastatic breast cancer [9], higher [^18^F]FDG uptake in the primary tumor tends to associate with worse OS. For PDAC, recent studies use a manifold of different metabolic parameters to correlate to OS, yielding conflicting results.

Some studies showed a significant association between standardized uptake value (SUV), mostly SUV_max_, and OS [10, 11], but results are equivocal [12, 13]. Measures for intratumoral heterogeneity may provide alternative biomarkers with prognostic impact [14, 15].

Several factors should be considered when metabolic parameters are assessed in PDAC. First, respiratory motion-related artifacts complicate the quantification of metabolic parameters. It is known that the movement of the diaphragm during image acquisition (with relatively long acquisition times, typically 3–4 min per bed position) causes motion artifacts, which results in blurring of anatomical borders, overestimation of lesion volume, and underestimation of radiotracer uptake in PET/CT-imaging [16–18]. Several methods of respiratory-gated imaging have been studied, including amplitude-based optimal respiratory gating (ORG) with HD Chest [18], resulting in significant decrease in lesion volume and increase in SUV_mean_ values when compared to non-gated imaging. Results changed more drastically for lesions closer to the diaphragm. So, evaluation of metabolic behavior of pancreatic lesions could gain accuracy using ORG.

Secondly, recent literature suggests that specific genetic mutations affect tumor metabolism [19]. For example, in colorectal cancer, KRAS-mutated tumors show increased SUV_max_ values as compared to KRAS wild-type tumors [20]. In prostate cancer, AKT1 activation was associated with accumulation of aerobic glycolysis metabolites, whereas MYC overexpression was associated with dysregulated lipid metabolism [21]. In parallel, PDAC tumors have a heterogeneous genetic mutation profile that might affect their metabolic phenotype [22–24]. Since intra-tumoral heterogeneity, as visualized and assessed on imaging studies, can play an important role in defining tumor biology [25], SUV_max_ or SUV_mean_ might, intrinsically to their physical definitions, not always be the most relevant parameter.

In this study, we analyzed a large cohort of patients with proven PDAC scanned with [^18^F]FDG PET/CT during diagnostic work-up. To investigate the impact of respiratory motion correction on the quantification of metabolic parameters, we analyzed ORG-gated [^18^F]FDG PET/CT and the corresponding standard [^18^F]FDG PET/CT for commonly used metabolic parameters, first and second-order texture features, and their correlation with OS.

## Materials and methods

### Study design and population

In this retrospective explorative study, data from all patients who underwent an [^18^F]FDG PET/CT scan at the Radboudumc in Nijmegen for suspected pancreatic malignancy between November 2004 and January 2015 were collected (Table 1). We also included patients who were scanned elsewhere and were referred for further diagnostic evaluation. The available histopathological data acquired by either biopsy or surgery were analyzed and 158 patients with histologically proven pancreatic ductal adenocarcinoma were identified (Additional file 1: Figure S1, CONSORT flowchart). In sixty-nine of these patients, an ORG reconstructed scan was available. We used this group to test the additional value of ORG scanning on the quantification of metabolic parameters. Both non-ORG (according to EANM guidelines) and ORG scans were correlated to OS. Given the retrospective nature of this study and the anonymized handling of data, informed consent was waived by the institutional review board (protocol CMO2018-4420). Scans were anonymized before review and the readers were unaware of the clinical or histopathological data.

**Table 1:** Patient characteristics

Characteristic	Number/value
Sex	
Male	42
Female	27
Age at PET/CT scan	
Mean (± SD)	65.17 ± 8.71
Median (range)	66 (40–82)
Survival after PET/CT scan (weeks)	
Mean (± SD)	64.75 ± 63,36
Median (range)	40 (1–253)
Injected dose of ^18^F-FDG (MBq/kg)	
Mean (± SD)	232.99 ± 54.40
Median (range)	231 (81–384)
Resectable tumors	31
Non-resectable tumor	38
Localization tumor	
Head	50
Body	15
Tail	4

### Image acquisition

All patients fasted for a minimum of 6 h prior to intravenous administration of [^18^F]FDG. Subsequently, patients rested for approximately 60 min before imaging.

Whole-body PET images were acquired using a Biograph 40 mCT (Siemens Medical Solutions, Knoxville Tennessee, USA) PET/CT scanner equipped with a dual-slice CT. PET images were reconstructed according to the EANM procedure guidelines for tumor PET imaging [26]. A TrueX algorithm was used with point spread function (PSF) and time of flight (TOF) measurements, using three iterations, 21 subsets, matrix size 200 × 200 (pixel spacing of 4.07 mm), full width half maximum (FWHM) of 3 mm, and using 2 min of PET data. Post-processing was performed using a 3D Gaussian filter kernel, 3.0 mm. For respiratory gating, we used an amplitude-based ORG algorithm, which is integrated in the Syngo 2012A MI.PET/CT software by Siemens, designated HD Chest. The procedures were performed as previously described [18]. In short, the main user input for the ORG algorithm is the percentage duty cycle, which is the percentage of the total acquired true coincidences used for image reconstruction. The ORG algorithm calculates an optimal amplitude range for a given duty cycle, by calculating the amplitude range for different values of the lower limit (*L*). With each value of *L*, the upper limit (*U*) is adjusted to include the specified percentage of the acquired PET data, and an amplitude range (*W*) is calculated through a simple subtraction (*U* − *L*). The optimal amplitude range is defined as the smallest amplitude range obtained and calculated by minimizing *W*.

The reconstruction settings were kept constant between ORG and non-ORG images; the percentage of duty cycles (total acquired true coincidences used for image reconstruction) was set at 35%, corresponding with 2 min of PET data.

Low-dose CT scans for attenuation corrections were reconstructed using a B19f convolution kernel, slice thickness 5.0 mm, and CT images for anatomical reference were reconstructed using a B31f convolution kernel, slice thickness 3.0 mm.

### Image analysis

All [^18^F]FDG PET/CT scans were evaluated using the Inveon Research Workspace 4.2 (Preclinical Solutions, Siemens Medical Solutions USA, Knoxville Tennessee, USA). After manual annotation of tumor region, a 40% SUV_max_ isocontour was used to delineate the metabolic tumor volume. Uptake of [^18^F]FDG projecting over adjacent structures, for example, uptake in the duodenum or biliary stent, was manually excluded from the VOIs, unless this was identified as tumor on contrast-enhanced CT (ceCT) or magnetic resonance cholangiopancreatography (MRCP). The first and second-order features were extracted using the PyRadiomics toolbox [27]. For the texture feature extraction, the images were discretized using a fixed number of 255 bins. The texture features were extracted from the co-occurrence (GLCM), gray-level run-length (GLRLM), and gray-level size-zone texture matrices (GLSZM). These matrices were generated by considering 26 connected voxels, 13 directions in three dimensions with a distance of 1 voxel, as described previously [27].

### Statistical analysis

Statistical analysis was performed using R Software. Quantification of metabolic parameters measured on non-ORG versus ORG scans was compared using the Wilcoxon paired *t* test (no Gaussian distribution assumed).

Optimal cutoff values were determined for each individual parameter on both ORG and non-ORG scans, based on Cox proportional hazard model and log-rank testing in Kaplan Meier survival curves. Overall survival of a patient was defined from the date of the PET/CT acquisition until the date of the patient’s death.

Univariate analysis of the data was performed on all 69 non-ORG and ORG scans using the Cox proportional hazard model, after scaling the features using the standard-scaler of Scikit-learn [28]. Additionally, all respiratory-gated scans were compared to their corresponding non-ORG counterpart to test the additional value of respiratory-gating on quantification of metabolic parameters.

## Results

### ORG improves the detection of pancreatic lesions and assessment of intra-tumoral heterogeneity

**Fig. 1:**
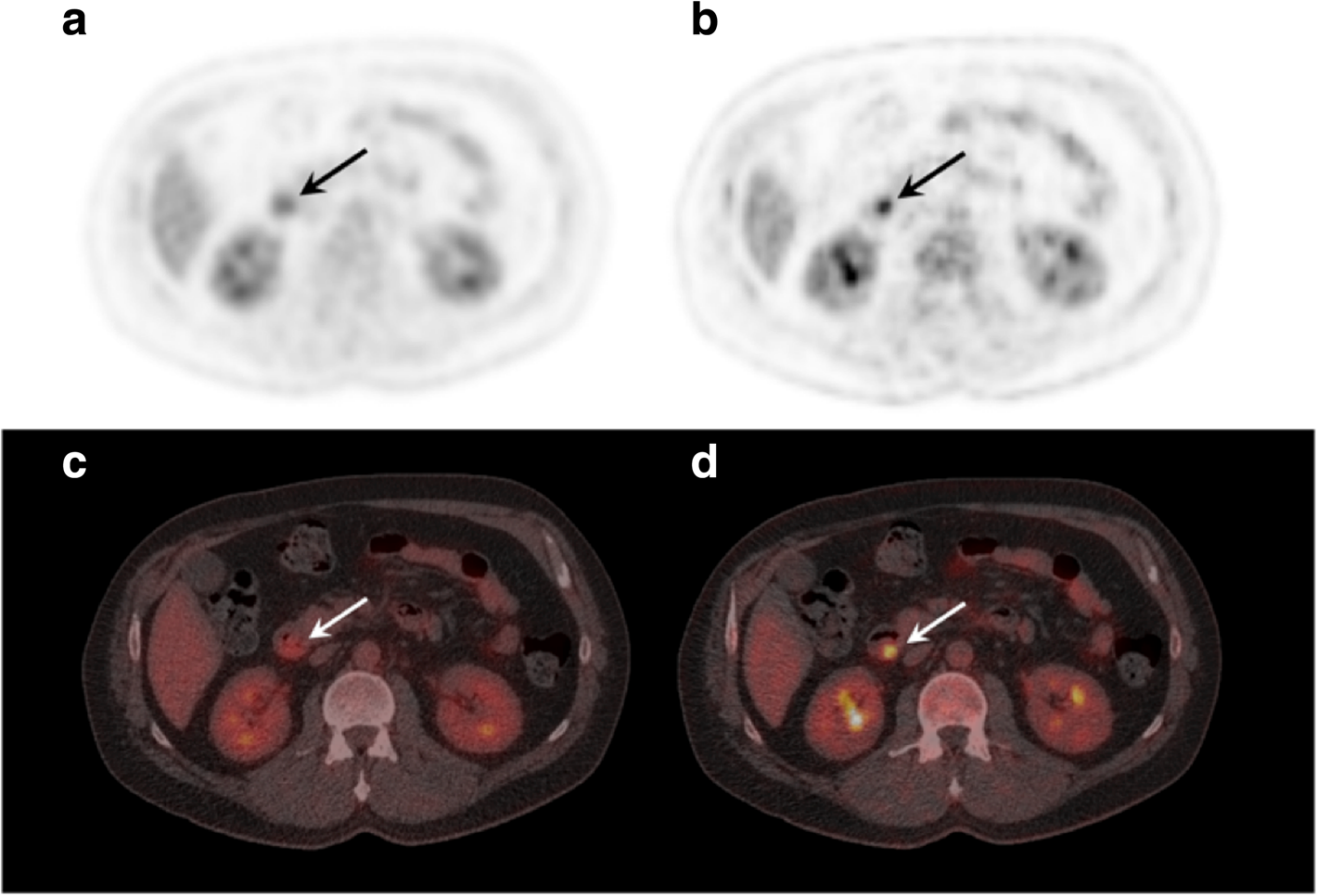
Optimal respiratory gating improves detection of small pancreatic and ampullary lesions. A [^18^F]FDG PET/CT scan reconstructed according to EANM guidelines (**a**, **c**) and with optimal respiratory gating (**b**, **d**), demonstrating markedly improvement of a small ampullary lesion in a 45-year old male who presented with jaundice. Endoscopic retrograde cholangiopancreatography (ERCP) showed a polyp-like lesion located at the ampulla of Vater, which was histologically characterized as metaplastic mucosa

Pancreatic lesions can present in a wide range of variations on [^18^F]FDG PET/CT, but commonly, ductal adenocarcinomas are FDG avid beyond the level of liver uptake, with corresponding hypodense lesions on ceCT. Smaller lesions can be overlooked as physiological [^18^F]FDG accumulation in the duodenum. Small size and low absolute level of [^18^F]FDG accumulation renders small lesions particularly prone to partial-volume effects and motion artifacts. Correction for respiratory motion artifacts improved the visual detection of small pancreatic ductal adenocarcinomas in the pancreatic head and ampullary carcinomas (Fig. 1).

**Fig. 2:**
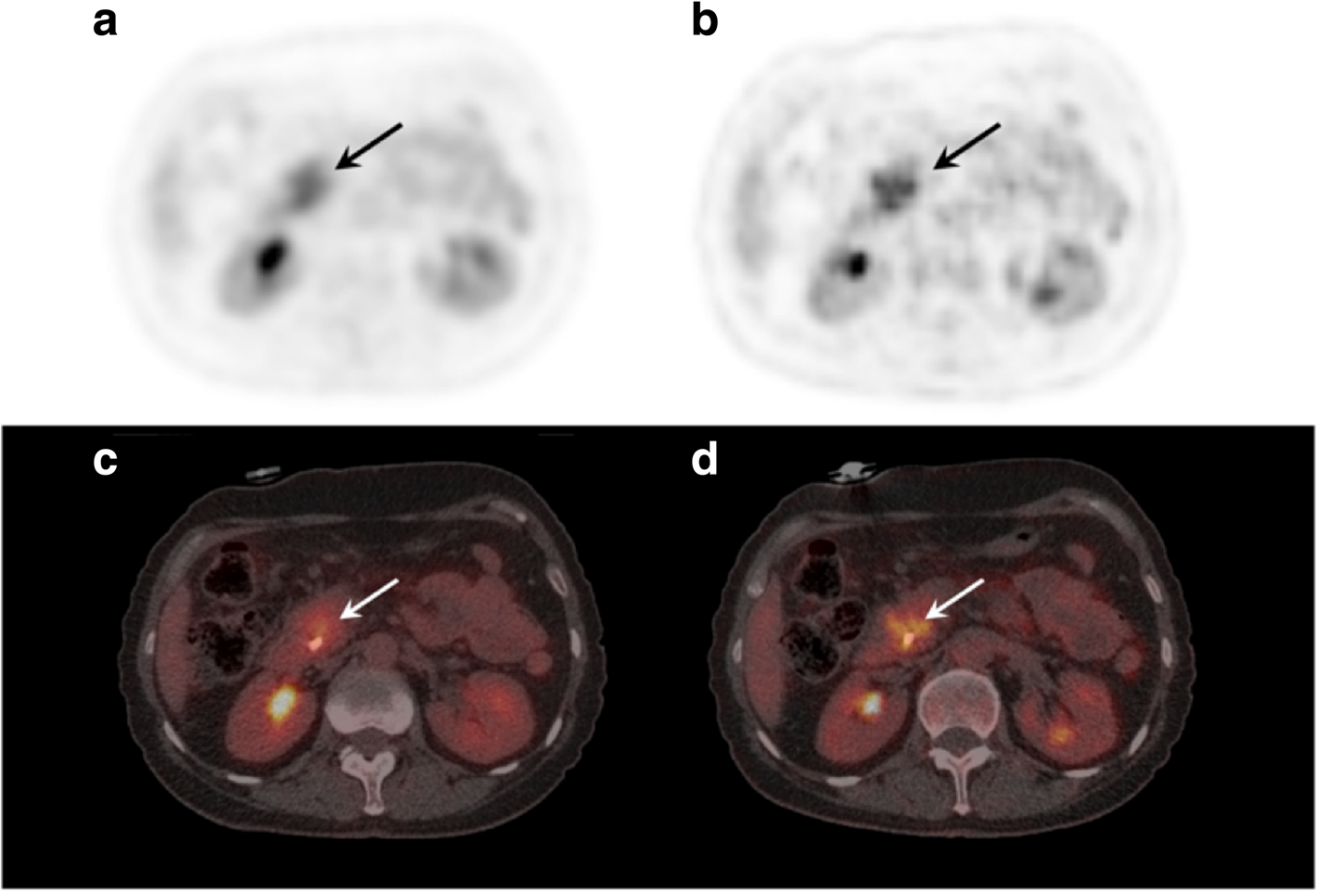
Optimal respiratory gating impacts assessment of intra-tumoral heterogeneity. A [^18^F]FDG PET/CT scan reconstructed according to EANM guidelines (**a**, **c**) and with optimal respiratory gating (**b**, **d**) showing changes in the heterogeneous pattern of intra-tumor [18F]FDG uptake

Using ORG reconstruction, the visual assessment of intra-tumoral regional differences in [^18^F]FDG accumulation changed, revealing regions of absent [^18^F]FDG accumulation or multiple foci of increased [^18^F]FDG accumulation (Fig. 2).

### ORG significantly impacts quantification of metabolic parameters

**Fig. 3:**
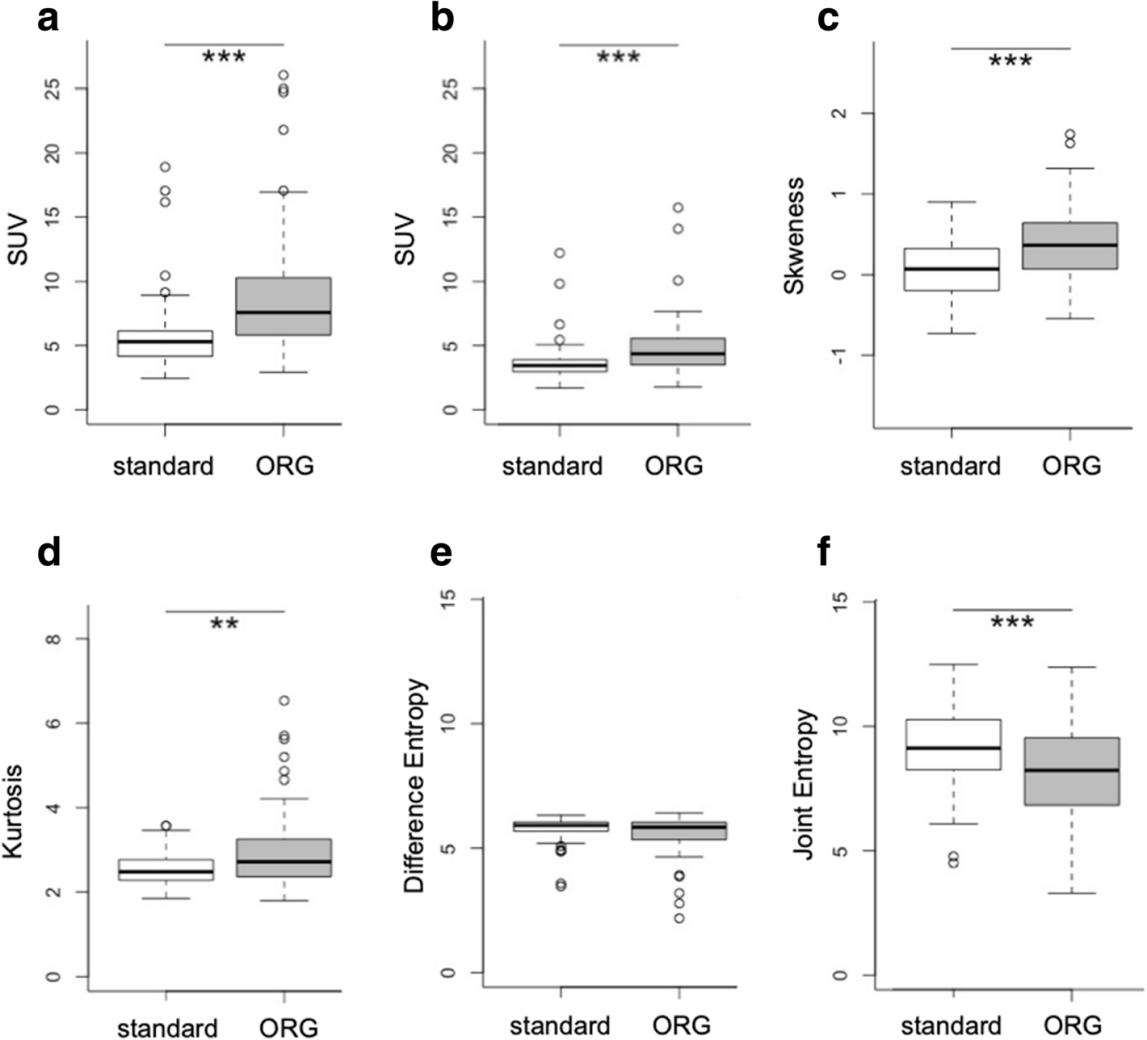
Optimal respiratory gating impacts the quantification of [^18^F]FDG PET-derived image parameters. The quantification of PET-derived parameters changes significantly after compensating for respiratory motion, as demonstrated for **a** SUV_max_, **b** SUV_mean_, **c** skewness, **d** Kurtosis, **e** difference entropy, and **f** joint entropy

Next, we compared pairs of standard and ORG reconstructed scans to explore the impact of respiratory motion on the quantification of metabolic parameters. In concordance with previous studies using respiratory gating techniques, the parameters SUV_max_, SUV_mean_, and SUV_diff_ showed significantly higher values on ORG scans compared to the corresponding non-ORG scans. The other metabolic parameters, e.g., MTV40%, significantly decreased (Additional file 2: Table S1, Fig. 3).

First-order features that describe intratumoral heterogeneity changed, for example, skewness, kurtosis, standard deviation, and entropy of the histogram increased significantly in the ORG reconstructed scan. Except for the features difference entropy and gray level variance, second-order features also showed a significant increase or decrease in the ORG reconstructed scans, for example, dissimilarity and joint entropy.

### Prognostic impact of metabolic parameters is affected by ORG

**Fig. 4:**
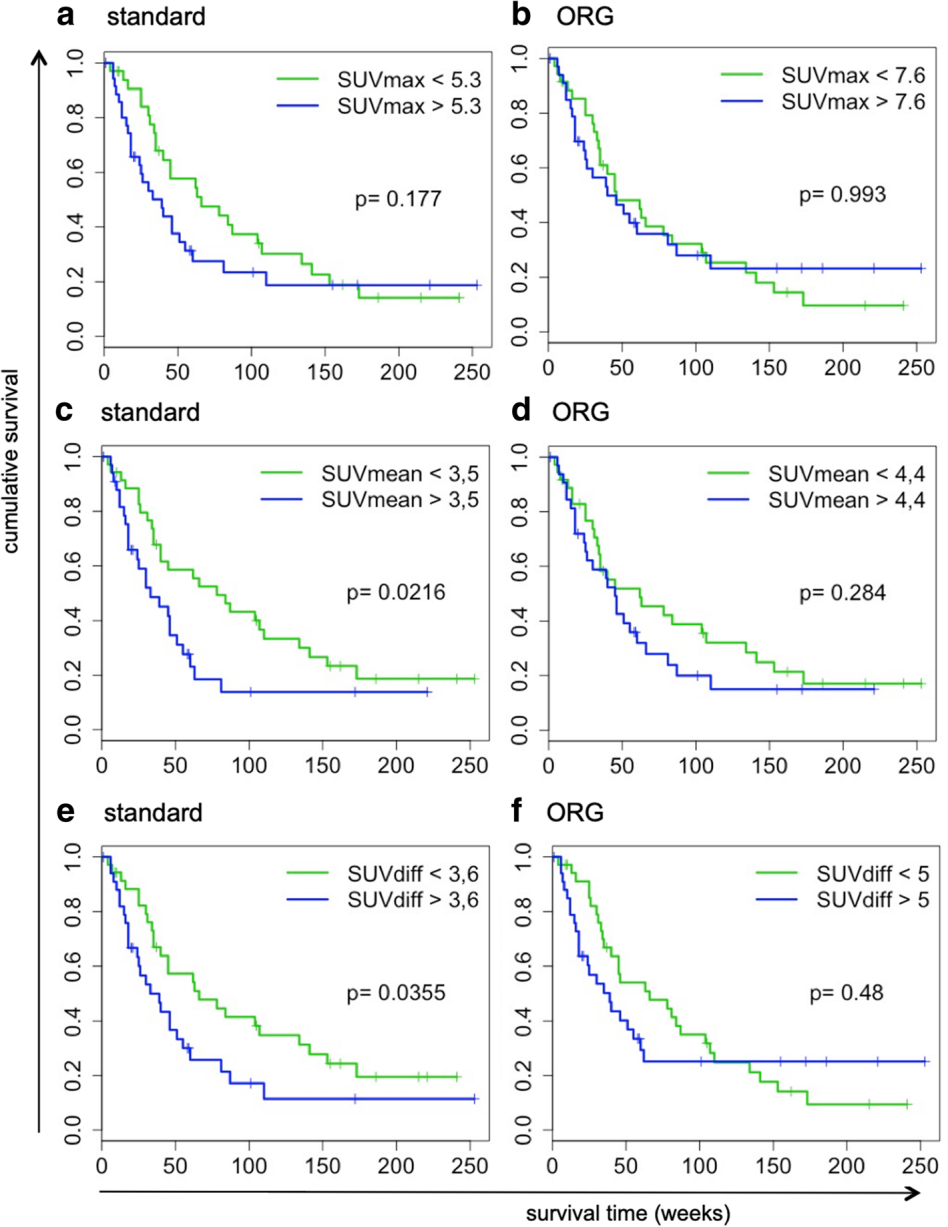
Optimal respiratory gating impacts the correlation of standard [^18^F]FDG PET-derived image parameters with overall survival. Kaplan-Meier survival curves demonstrating the impact of compensation of respiratory motion on the correlation of PET-derived parameters with overall survival for standard PET-derived parameters, **a**–**b** SUV_max_, **c**–**d** SUV_mean_, and **e**–**f** SUV_diff_

**Fig. 5:**
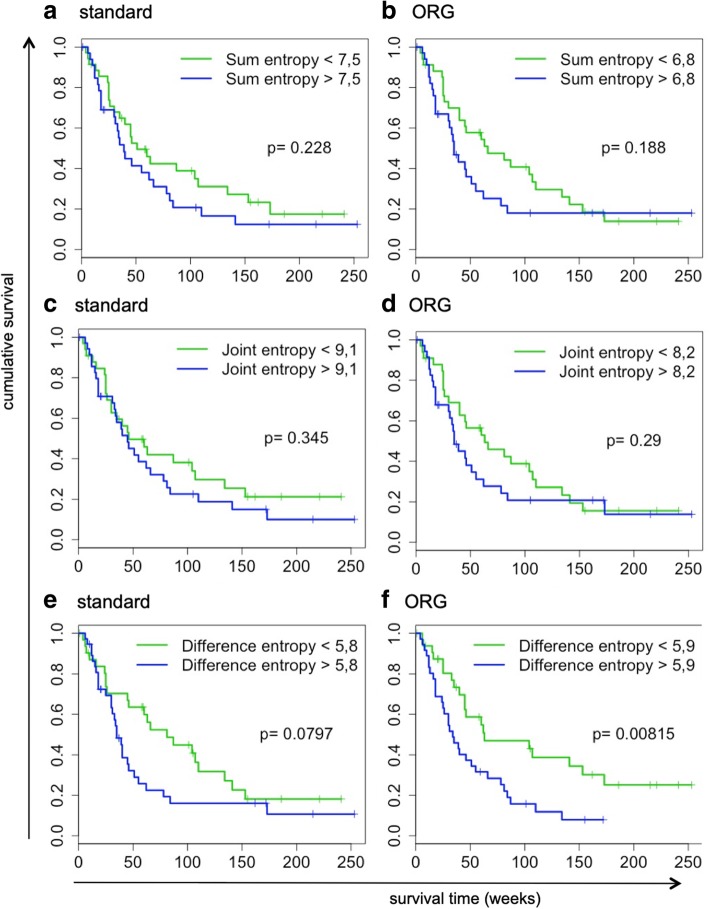
Optimal respiratory gating impacts the correlation of [^18^F]FDG PET-derived texture features with overall survival. Kaplan-Meier survival curves demonstrating the impact of compensation of respiratory motion on the correlation of PET-derived parameters with overall survival for PET-derived texture features, **a**–**b** sum entropy, **c**–**d** joint entropy, and **e**–**f** difference entropy

As the quantification of metabolic parameters changed considerably using ORG, we investigated whether their correlation with OS was consequently affected. For both standard and ORG reconstructed scans, optimal cutoff values for correlation with OS were defined. Logistic regression analysis demonstrated that the correlation with OS of most metabolic parameters changed considerably, but in an inconsistent pattern (Table 2). Standard PET-derived parameters SUV_max_, SUV_mean_, and SUV_diff_ were no longer associated with OS after ORG reconstruction (Table 2, Fig. 4), whereas parameters that describe heterogeneity (sum entropy) became significant, or borderline significant (difference entropy, joint entropy), associated with OS (Table 2, Fig. 5).

**Table 2:** Optimal respiratory gating impacts the correlation of [^18^F]FDG PET-derived image features with overall survival, assessed by univariate logistic regression analysis

Image feature	Non-ORG			ORG		
	HR	95%CI	*p* value	HR	95%CI	*p* value
MTV	1.056	(0.636–1.751)	0.834	1.218	(0.638–2.322)	0.550
SUV_min_	1.206	(0.964–1.510)	0.102	0.976	(0.756–1.258)	0.850
SUV_max_	1.324	(1.031–1.700)	0.026	1.121	(0.860–1.461)	0.397
SUV_mean_	1.328	(1.072–1.645)	0.008	1.137	(0.891–1.451)	0.303
SUV_diff_	1.360	(1.011–1.830)	0.041	1.155	(0.884–1.510)	0.288
Variance	1.242	(0.965 –1.598)	0.080	1.045	(0.792–1.378)	0.756
Skewness	1.088	(0.843–1.404)	0.516	0.770	(0.577–1.027)	0.076
Kurtosis	0.871	(0.668–1.135)	0.306	0.894	(0.663–1.206)	0.462
Entropy.image	1.232	(0.910–1.669)	0.177	1.198	(0.910–1.577)	0.196
STD	1.271	(0.968–1.669)	0.083	1.064	(0.812–1.395)	0.653
Energy	0.999	(0.769–1.298)	0.992	0.812	(0.594–1.110)	0.181
Contrast	1.051	(0.813–1.359)	0.704	0.809	(0.612–1.069)	0.135
Dissimilarity	1.056	(0.812–1.374)	0.684	0.845	(0.650–1.099)	0.208
Homogeneity.1	0.917	(0.680–1.237)	0.571	1.018	(0.793–1.309)	0.887
Homogeneity.2	0.899	(0.652–1.241)	0.517	0.982	(0.766–1.258)	0.884
Correlation	0.923	(0.724–1.178)	0.521	1.181	(0.899–1.552)	0.231
Difference Entropy.1	1.131	(0.828–1.543)	0.439	1.362	(0.989–1.876)	0.055
Joint Entropy.2	1.093	(0.824–1.450)	0.535	1.290	(0.980–1.698)	0.068
Sum Entropy.3	1.086	(0.819–1.439)	0.568	1.340	(1001–1.795)	0.048
Short.Run.Emphasis	1.097	(0.799–1.506)	0.565	1.040	(0.804–1.346)	0.764
Long.Run.Emphasis	0.914	(0.654–1.278)	0.599	0.923	(0.702–1.214)	0.567
Gray.Level.Non-Uniformity	0.983	(0.590–1.639)	0.948	0.967	(0.503–1.860)	0.919
Gray.Level.Non-Uniformity.Normalized	0.976	(0.746–1.278)	0.861	0.766	(0.552–1.062)	0.101
Run.Length.Non-Uniformity	1.059	(0.650–1.727)	0.817	1.244	(0.670–2.310)	0.489
Run.Length.Non-Uniformity.Normalized	1.094	(0.800–1.496)	0.575	1.033	(0.800–1.334)	0.804
Run.Percentage	1.091	(0.789–1.508)	0.598	1.057	(0.812–1.374)	0.681
GrayLevelVariance	0.971	(0.744–1.268)	0.832	0.873	(0.665–1.145)	0.325
SmallAreaHighGrayLevelEmphasis	1.034	(0.781–1.367)	0.818	1.224	(0.924–1.622)	0.158
GrayLevelNonUniformityNormalized	0.982	(0.752–1.283)	0.895	0.773	(0.559–1.067)	0.110
SizeZoneNonUniformityNormalized	1.082	(0.824–1.420)	0.572	0.876	(0.670–1.145)	0.331
SizeZoneNonUniformity	1.098	(0.740–1.629)	0.643	1.310	(0.796–2.156)	0.286
GrayLevelNonUniformity	1.011	(0.625–1.637)	0.964	1.063	(0.585–1.932)	0.841
LargeAreaEmphasis	0.831	(0.447–1.543)	0.557	0.726	(0.400–1.320)	0.254
ZoneVariance	0.733	(0.262–2.053)	0.555	0.611	(0.240–1.556)	0.198
ZonePercentage	1.078	(0.801–1.451)	0.621	0.963	(0.747–1.241)	0.769

## Discussion

Current literature shows equivocal results for the correlation of metabolic parameters measured by [^18^F]FDG PET/CT and survival in patients with pancreatic cancer. In this study, we retrospectively analyzed a cohort of histologically proven PDAC patients to (1) study the impact of optimal respiratory gating on the quantification of the most commonly used metabolic parameters and (2) assess whether the correlation of these metabolic parameters and OS is affected.

In a relatively large cohort of patients, we noted that ORG results in the improved visibility of especially smaller sized pancreatic lesions, together with better visual assessment of intratumoral heterogeneity. Furthermore, we demonstrated that ORG imaging has a significant impact on quantification of all measured metabolic parameters, including the commonly used SUV_max_ and SUV_mean_, and also first- and second-order texture features that describe intratumoral heterogeneity. As expected, ORG imaging more accurately allocated measured activity to the corresponding voxel and therefore resulted in smaller measured tumor volumes with a wider range of values. Importantly, the correlation of metabolic parameters with OS significantly changed using ORG; some parameters did now correlate whereas other parameters did no longer correlate with OS.

Recent studies on metabolic parameters and their predictive value in PDAC have yielded equivocal findings [10, 12, 13, 29], and no single parameter was confirmed to predict behavior of PDAC. The uncertainties in these observations can be addressed from different angles; first, tumor metabolism is only one feature of a highly complex tumor microenvironment that involves a multitude of different functional phenotypes [30]. Other hallmarks of cancer than tumor metabolism perhaps are more critical to clinical outcome. Secondly, PDAC are generally characterized by high stromal content, which impacts pathways of tumor metabolism. Other metabolic processes, e.g., glutaminolysis or autophagy, are also involved to different degrees [19]. This might in part be reflected by heterogeneous patterns of glucose metabolism and renders a single PET parameter less relevant in this cancer type. Our study might help to explain the contradictory results from a technical standpoint, as it illustrates the difficulties with accurate quantification of metabolic parameters in organs just below the diaphragm and subjected to respiratory motion during acquisition.

The impact of ORG on the prognostic impact of PET-derived metabolic parameters or clinical decision-making was beyond the scope of this study and in general involves more information than the metabolic parameters investigated here. The retrospective nature of this study and the cohort size does not allow a comprehensive multivariate analysis to investigate whether ORG-gated measurements result in improved prognostic impact. Further prospective studies are needed to establish whether [^18^F]FDG PET imaging has a prognostic role and which (set of) parameters are most accurate. Furthermore, it is highly interesting to investigate in further studies whether, for example, detection of metastases in liver and lymph nodes or local tumor growth is impacted by ORG. Moreover, we investigated a homogeneous cohort with histologically proven PDAC to rule out variations by different etiologies, whereas in the clinical setting, patients with (peri-) ampullary lesions or (focal) pancreatitis will also be referred for [^18^F]FDG PET/CT imaging under the suspicion of pancreatic cancer.

Although correction for respiratory motion impacts the quantification of metabolic parameters derived from PET imaging, we could not demonstrate a consistent pattern in correlation with overall survival. Given the heterogeneous nature of cancer, it can be hypothesized that metabolic parameters that assess intratumoral heterogeneity better classify the underlying tumor biology than single-voxel parameters like SUV_max_ or averaged parameters like SUV_mean_ or TLG. Studies in esophagus [31], breast [32] and other cancers [33, 34] support this hypothesis and illustrate the potential of PET texture features to predict survival and treatment outcome. However, extracting texture features from PET images is not standardized and subject to variations in image reconstruction, tumor delineation, SUV normalization, and SUV discretization [35–37]. In this study, we adhered to the 40% SUV_max_ threshold for tumor delineation which is widely accepted and recommended by the EANM guidelines [38]. This isocontour corresponds best with the actual dimensions of the tumor in non-ORG images. As there is no gold standard as to assess the actual tumor volume, e.g., CT or MRCP, and anatomical imaging was not standardized in this cohort, our data does not allow cross-validation of this tumor delineation method for ORG scans.

A recent study in a similar cohort of patients with PDAC suggests that first-order entropy is an independent predictor for 2-year survival [14]. As texture features are increasingly being reported to correlate with gene expression profiles [39], it is tempting to speculate that ORG imaging further improves the assessment of intratumoral heterogeneity using advanced image analyses and facilitates in vivo classification of tumor behavior in relevant clinical settings [40].

## Conclusions

The present study demonstrates that correction for respiratory motion artifacts significantly changes the quantitative measurement of metabolic parameters, including first- and second-order measures for intratumoral heterogeneity, in primary pancreatic ductal adenocarcinoma, with direct impact on their correlation with overall survival.

## Additional files

Additional file 1: **Figure S1.** CONSORT flow chart. (PNG 137 kb)

Additional file 2: **Table S1.** Optimal respiratory gating impacts the quantification of [^18^F]FDG PET-derived image features. (DOCX 23 kb)

## Abbreviations

[^18^F]FDG: 2′-Deoxy-2′-[^18^F]fluoro-D-glucose
ceCT: Contrast-enhanced computed tomography
EANM: European Association for Nuclear Medicine
ERCP: Endoscopic retrograde cholangiopancreatography
FWHM: Full width half maximum
MRCP: Magnetic resonance cholangiopancreatography
ORG: Optimal respiratory gating
OS: Overall survival
PDAC: Pancreatic ductal adenocarcinoma
PET: Positron emission tomography
PSF: Point spread function
SUV: Standardized uptake value
TLG: Total lesion glycolysis
TOF: Time of flight
