# Supplementary figures and images for: Optimal respiratory-gated [18F]FDG PET/CT significantly impacts the quantification of metabolic parameters and their correlation with overall survival in patients with pancreatic ductal adenocarcinoma
Source: EJNMMI Res. 2019 Mar 13;9:24. doi: 10.1186/s13550-019-0492-y (PMC6419652; doi:10.1186/s13550-019-0492-y)

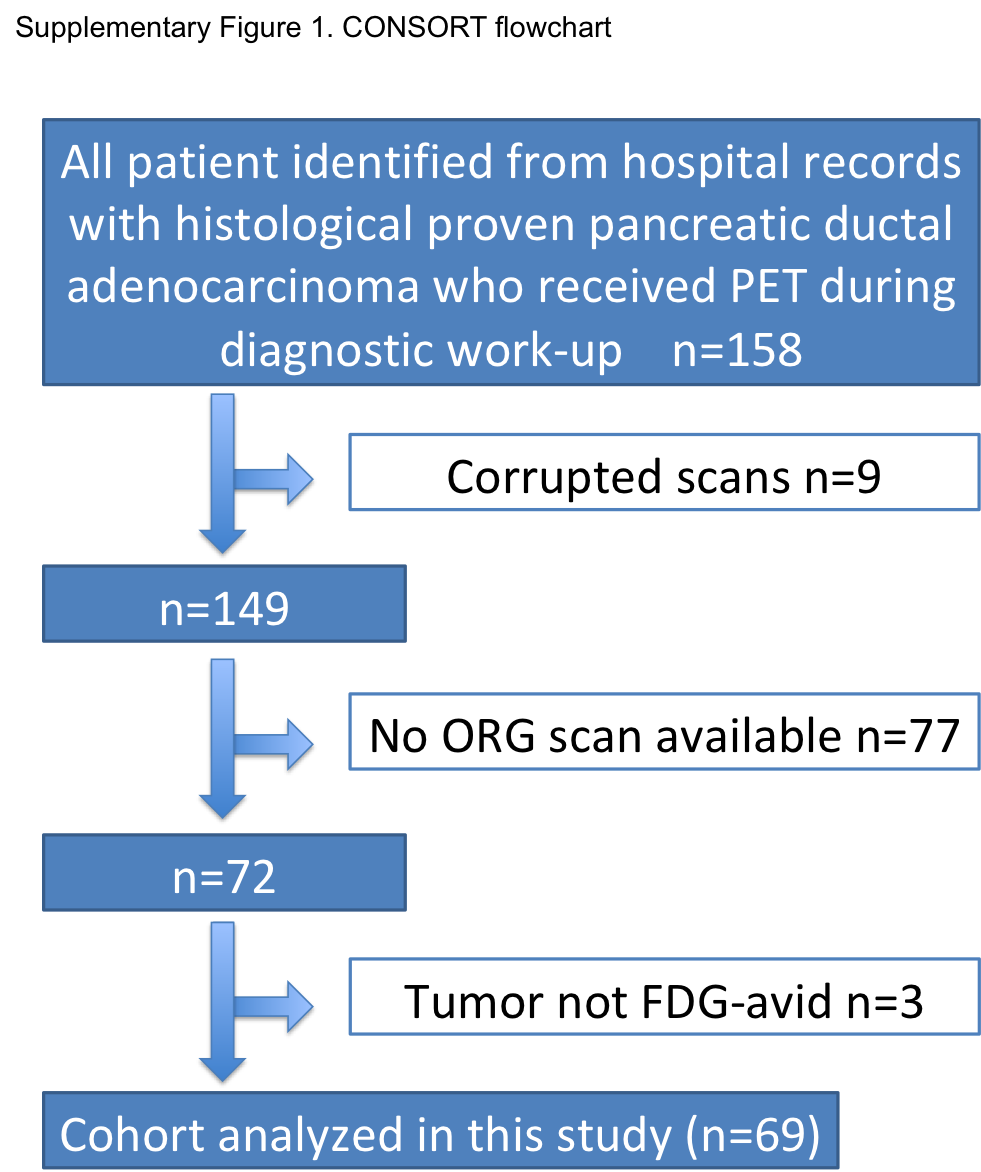

Supplement: Supplementary file 1 — Figure S1. CONSORT flow chart. (PNG 137 kb) [file 13550_2019_492_MOESM1_ESM.png]
