# Supplementary material for: Optimal respiratory-gated [18F]FDG PET/CT significantly impacts the quantification of metabolic parameters and their correlation with overall survival in patients with pancreatic ductal adenocarcinoma
Source: EJNMMI Res. 2019 Mar 13;9:24. doi: 10.1186/s13550-019-0492-y (PMC6419652; doi:10.1186/s13550-019-0492-y)
Supplement: Supplementary file 2 — Table S1. Optimal respiratory gating impacts the quantification of [18F]FDG PET-derived image features. (DOCX 23 kb) [file 13550_2019_492_MOESM2_ESM.docx]

**Supplementary Tabel 1.** Optimal respiratory gating impacts the quantification of [^18^F]FDG PET derived image features

| **Image feature** | **non-ORG** | | **ORG** | |  |
| --- | --- | --- | --- | --- | --- |
|  | **mean** | **std** | **mean** | **std** | **p-value** |
| MTV40% | 754.493 | 1.282.598 | 597.087 | 1.379.566 | <0.00001 |
| SUVmin | 1.898 | 0.751 | 2.247 | 1.595 | 0.31228 |
| SUVmax | 5.876 | 2.998 | 8.891 | 4.89 | <0.00001 |
| SUVmean | 3.717 | 1.552 | 4.832 | 2.316 | <0.00001 |
| SUVdiff | 3.977 | 2.785 | 6.643 | 4.502 | <0.00001 |
| Variance | 1.071 | 2.097 | 2.523 | 3.232 | <0.00001 |
| Skewness | 0.078 | 0.375 | 0.383 | 0.451 | <0.00001 |
| Kurtosis | 2.55 | 0.398 | 2.977 | 0.938 | 0.00013 |
| Entropy.image | 1.67 | 0.7 | 2.217 | 0.707 | <0.00001 |
| STD | 0.852 | 0.592 | 1.356 | 0.834 | <0.00001 |
| Energy | 0.004 | 0.007 | 0.009 | 0.017 | <0.00001 |
| Contrast | 2.040.957 | 1.243.718 | 3.062.087 | 2.174.004 | <0.00001 |
| Dissimilarity | 34.863 | 10.486 | 41.402 | 14.6 | <0.00001 |
| Homogeneity.1 | 0.077 | 0.02 | 0.071 | 0.025 | 0.00048 |
| Homogeneity.2 | 0.032 | 0.012 | 0.029 | 0.017 | 0.00388 |
| Correlation | 0.675 | 0.151 | 0.492 | 0.229 | <0.00001 |
| Difference Entropy.1 | 5.778 | 0.513 | 5.546 | 0.814 | 0.20496 |
| Joint Entropy.2 | 9.081 | 1.665 | 8.223 | 1.959 | <0.00001 |
| Sum Entropy.3 | 7.21 | 1.057 | 6.521 | 1.354 | <0.00001 |
| Short.Run.Emphasis | 0.994 | 0.003 | 0.995 | 0.004 | 0.00216 |
| Long.Run.Emphasis | 1.025 | 0.013 | 1.022 | 0.021 | 0.0018 |
| Gray.Level.Non-Uniformity | 5.41 | 8.66 | 5.098 | 11.014 | 0.00083 |
| Gray.Level.Non-Uniformity.Normalized | 0.01 | 0.006 | 0.013 | 0.01 | <0.00001 |
| Run.Length.Non-Uniformity | 724.69 | 1.189.957 | 568.831 | 1.259.814 | <0.00001 |
| Run.Length.Non-Uniformity.Normalized | 0.984 | 0.008 | 0.986 | 0.011 | 0.00192 |
| Run.Percentage | 0.992 | 0.004 | 0.993 | 0.006 | 0.0017 |
| GrayLevelVariance | 3.088.222 | 769.455 | 2.913.204 | 953.713 | 0.06212 |
| SmallAreaHighGrayLevelEmphasis | 17.039.216 | 4192.22 | 14.041.383 | 4.507.911 | <0.00001 |
| GrayLevelNonUniformityNormalized | 0.01 | 0.006 | 0.013 | 0.01 | <0.00001 |
| SizeZoneNonUniformityNormalized | 0.831 | 0.064 | 0.857 | 0.07 | 0.00064 |
| SizeZoneNonUniformity | 489.041 | 622.884 | 384.314 | 662.878 | 1,00E-05 |
| GrayLevelNonUniformity | 4.58 | 6.711 | 4.239 | 8.217 | 0.00029 |
| LargeAreaEmphasis | 1.417 | 0.448 | 1.503 | 1.259 | 0.00177 |
| ZoneVariance | 0.177 | 0.326 | 0.296 | 1.12 | 0.00166 |
| ZonePercentage | 0.902 | 0.045 | 0.916 | 0.053 | 0.0013 |
